# Microwave Assisted Synthesis and Biological Activity of Novel Coumarinyltriazolothiadiazoles

**DOI:** 10.4103/0250-474X.70483

**Published:** 2010

**Authors:** P. Manoj Kumar, T. K. Ravi, R. Chawla, S. Bhuvana, G. Sonia, S. Gopalakrishnan

**Affiliations:** Department of Pharmaceutical Chemistry, College of Pharmacy, Sri Ramakrishna Institute of Paramedical Sciences, 395, Sarojini Naidu Road, Coimbatore-641 044, India

**Keywords:** Antimicrobial, coumarin, thiadiazole, Schiff’s base, triazole

## Abstract

A series of new 3-(4-methylcoumarinyl-7-oxymethyl)-6-substitutedphenyl-5,6-dihydro-s-triazolo (3,4-b)(1,3,4)-thiadiazoles 2(a-j) have been synthesized by reacting 5-(4-methyl coumarinyl-7-oxymethyl)-4-amino-3-mercapto(4H)-1,2,4-triazole with various aromatic aldehydes by microwave assisted organic synthesis. The structure of the compounds 2 (a-j) has been confirmed by IR, ^1^H NMR and mass spectral data. All the compounds were screened for antimicrobial and antioxidant activity. Among the compounds tested, compounds 2d (4-dimethyl amino phenyl derivative) and 2h (3,4-dimethoxy phenyl derivative) showed better antimicrobial and antioxidant activity than rest of the compounds in the series.

Coumarins possess antimicrobial[1,2], antiviral[3], antipyretic[4], antiinflammatory[5] and antioxidant activities[1,2]. Triazolothiadiazoles have been reported to be associated with various biological activities such as antibacterial[1], antiinflammatory[6], analgesic[7] and CNS depressant[8] activity. In the light of these interesting biological activities, we have attempted to synthesize some new triazolothiadiazole derivatives bearing coumarin moiety and test the new compounds for antibacterial, antifungal and antioxidant activity. In our earlier work[2] we had reported the synthesis of 5-(4-methyl coumarinyl-7-oxymethyl)-4-amino-3-mercapto (4*H*)- 1, 2, 4 -triazole (1). In continuation of the previous work, certain 3-(4-methylcoumarinyl-7-oxymethyl)-6-substitutedphenyl-5,6-dihydro-*s*-triazolo (3,4-b) (1, 3, 4)-thiadiazoles 2(a-j) have been synthesized by reacting 5-(4-methyl coumarinyl-7-oxymethyl)-4-amino-3-mercapto (4*H*)- 1, 2, 4 -triazole with various aromatic aldehydes. For the present synthetic work we had opted for microwave irradiation technique, since our previous synthetic work[1,9] using microwave irradiation could bring about a remarkable reduction in the reaction time and gave increased yield of products when compared to conventional heating methods of synthesis. The structures of the synthesized compounds were assigned on the basis of IR, ^1^H NMR and mass spectral data. The synthesized compounds have been screened for their *in vitro* antimicrobial, antifungal and antioxidant activity.

Melting points were determined in open capillary tubes and are uncorrected. All the chemicals and solvents used were of laboratory grade and solvents were purified by suitable methods. IR spectra (KBr, cm^-1^) were recorded on a Jasco FT/IR-410 spectrometer. ^1^H NMR spectra was recorded in DMSO-d_6_using TMS as internal standard at IICT, Hyderabad, India. Mass spectra were recorded at IIT Chennai, India. Reactions were carried out in a Daewoo KOG-370A domestic microwave oven at 2450 MHz. The purity of the products was checked using TLC (Silica Gel-G, Merck).

As shown in Scheme 1, the synthesis of 3-(4-methylcoumarinyl-7-oxymethyl)-6-substitutedphenyl-5,6-dihydro-s-triazolo (3,4-b) (1,3,4)-thiadiazoles[1] 2(a-j) was carried out by taking a mixture of 5-(4-methyl coumarinyl-7-oxymethyl)-4-amino-3-mercapto (4*H*)-1,2,4-triazole (1) (3.04 g, 0.01 mol), substituted benzaldehyde (0.01 mol), p-toluene sulphonic acid (30 mg) and DMF (15 ml) in 100 ml borosil beaker, which was zapped inside a microwave oven for period of 3-4 min at 100% power. The reaction mixture was cooled and poured over ice cold water. The product so obtained was filtered, washed with water, dried and recrystallized using dimethyl formamide. The purity was established by a single spot on TLC plate. The solvent system used was chloroform:acetone (5:5). The characterization data of synthesized compounds are given in Table 1.

**TABLE 1:** CHARACTERIZATION DATA OF THE SYNTHESISED COMPOUNDS

Compound	R	Molecular formula	Molecular weight	Yield %	Melting point	R_f_#Value
2a	H	C_20_H_16_N_4_O_3_S	392	97	220	0.77
2b	3-NO_2_	C_20_H_15_N_5_O_5_S	437	88	270	0.81
2c	3, 4, 5-(OCH_3_)_3_	C_23_H_22_N_4_O_6_S	482	72	140	0.73
2d	4-NH (CH_3_)_2_	C_22_H_22_N_5_O_3_S	436	91	226	0.79
2e	4-OH	C_20_H_16_N_4_O_4_S	408	98	230	0.80
2f	4-Cl	C_20_H_15_N_4_O_3_SCl	426	71	250	0.74
2g	4-CH_3_	C_21_H_18_N_4_O_3_S	406	64	240	0.82
2h	3, 4- (OCH_3_)_2_	C_22_H_20_N_4_O_5_S	452	93	98	0.69
2i	4- OH, 3-OCH_3_	C_21_H_18_N_4_O_5_S	438	81	105	0.76
2j	4- OH, 3-OC_2_H_5_	C_22_H_20_N_4_O_5_S	452	54	215	0.83

# Solvent system used for TLC is benzene:methanol (9:1)

**Scheme 1:**
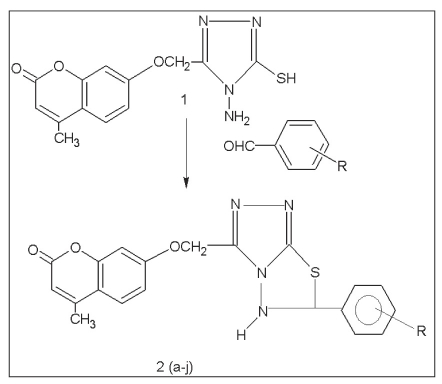
Synthesis of coumarinyltriazolothiadiazoles General method of synthesis of 3-(4-methylcoumarinyl-7-oxymethyl)-6-substitutedphenyl-5, 6-dihydro-s-triazolo (3, 4-b) (1, 3, 4)-thiadiazoles 2(a-j)

5b: IR (KBr)cm^-1^: 3434 (N-H), 1702 (C=O), 1606 (C=C),1519 (C-NO_2_), 1268 (C-O-C); 728 (N-C-S), ^1^H NMR (DMSO-d_6_) δ ppm: 3.4 (s, 3H, CH_3_), 5.4 (s, 2H, CH_2_), 6.9-7.1 (d, 1H, CH-thiadiazole), 7.2-8.6 (m, 8H, Ar-H), 8.6 (d, 1H, NH). Mass (m/z): 436 (M-1)^+^, 422, 391, 149.

Antimicrobial activity was determined using disc diffusion method[10] by measuring the inhibition zone in mm. All the compounds 2(a-j) were screened in vitro for antibacterial activity against *Staphylococcus aureus* and *Escherichia coli* and antifungal activity against *Candida albicans* at 500 μg/ml. Standard antibacterial drug ciprofloxacin and antifungal drug fluconazole at 10 μg/disc were also tested under similar conditions and these compounds showed highest activity (23-28 mm) against all these organisms tested. The data of compounds with their activity are given in Table 2.

The compounds synthesized were evaluated for antioxidant activity and compared with standard drug (ascorbic acid). The activity was evaluated using the DPPH method[9]. One millilitre of 0.3 mM DPPH ethanol solution was added to 2.5 ml of sample solutions of different concentrations (2, 4, 6, 8 and 10 μg/ml) and allowed to react at room temperature. After 30 min the absorbance values were measured at 518 nm and converted into the percentage antioxidant activity (AA) using the formula, AA% = 100–(Abs_sample_–Abs_blank_)×100/Abs_control_).

Ethanol (1.0 ml) plus drug solution (2.5 ml) was used as a blank. DPPH solution (1.0 ml, 0.3 mM) plus ethanol (2.5 ml) was used as a negative control. The positive controls were those using the standard solution (ascorbic acid). The IC_50_ values were calculated by linear regression plots, where abscissa represented the concentration of test drug solution (2, 4, 6, 8 and 10 μg/ml) and ordinate the average percent of antioxidant activity from three separate tests. The results are tabulated in Table 2.

**TABLE 2:** ANTIMICROBIAL AND ANTIOXIDANT ACTIVITY DATA OF THE SYNTHESISED COMPOUNDS

Compd.	Antibacterial activity		Antifungal activity	Antioxidant activity
	*S. aureus*	*E. coli*	*C. albicans*	IC_50_ μg/ml#
2a	-	-	-	7.6
2b	++	+++	++	7.7
2c	-	++	-	-
2d	++	++	-	5.7
2e	-	++	-	9.2
2f	++	++	-	8.2
2g	-	-	-	7.5
2h	-	-	++	5.6
2i	-	-	-	9.6
2j	-	-	++	-
Ciprofloxacin	++++	++++	----	----
Fluconazole	----	----	++++	----
Ascorbic acid	----	----	----	5.4

Inhibition diameter in mm: (-)μ6, (+)7-9, (++)10-15, (+++)16-22, (++++)23-28, (----) no activity.

# Values represent the average concentration required for exerting 50% of antioxidant activity from three separate tests.

All compounds were in conformity with the structures envisaged. The structures were proved on the basis of spectral data. Among the newly synthesized compounds showing antibacterial and antifungal activity, it was observed that compound 2b (3-nitrophenyl derivative) showed highest degree of antibacterial activity against Staphylococcus *aureus* and *Escherichia coli*. Compounds 2b (3-nitrophenyl derivative), 2h (3,4-dimethoxyphenyl derivative) and 2j (4-hydroxy-3-ethoxyphenyl derivative) showed better antifungal activity than rest of the newly synthesised compounds. Compounds 2d (4-dimethylaminophenyl derivative) and 2f (4-chlorophenyl derivative) showed moderate activity against *Staphylococcus aureus* and *Escherichia coli*. Rest of the compounds showed either minimal activity or no antimicrobial activity. In antioxidant studies, compounds 2d (IC_50_-5.7 μg/ml) (4-dimethylaminophenyl derivative) and 2h (IC_50_-5.6 μg/ml) (3,4-dimethoxyphenyl derivative) were identified to be more potent than rest of the compounds. From the results of biological screening we could conclude that 3,4-dimethoxyphenyl substituent and 4-N,N-dimethylphenyl substituent at position 6 of triazolothiadiazole are vital in improving the scavenging capacity of free radicals as evident from the antioxidant activity of compounds 2h and 2d, respectively which was comparable to that of standard ascorbic acid. Similarly the results of antimicrobial screening showed that substitution of 3-nitrophenyl substituent at position 6 of triazolothiadiazole confers both antibacterial and antifungal activity as seen with compound 2b. The compounds 2d and 2h can be chosen as lead moieties for antimicrobial and antioxidant studies and compounds 2b, 2j and 2f for antimicrobial studies.
